# Supplementary material for: Rapid turnover of life-cycle-related genes in the brown algae
Source: Genome Biol. 2019 Feb 14;20:35. doi: 10.1186/s13059-019-1630-6 (PMC6374913; doi:10.1186/s13059-019-1630-6)
Supplement: Supplementary file 3 — Protein alignments, corresponding Gblocks html file and CDS sequences for the lineage conserved gametophyte-biased genes. (ZIP 2109 kb) [file 13059_2019_1630_MOESM3_ESM.zip › Con_GA_alignments/Ec-06_004540.aa_ali.fasta-gb.htm]

Ec-06\_004540.aa.fasta.trans.aa\_ali.fasta


## Gblocks 0.91b Results

Processed file: **Ec-06\_004540.aa.fasta.trans.aa\_ali.fasta**  
Number of sequences: **2**  
Alignment assumed to be: **Protein**  
New number of positions: **253** (selected positions are underlined in blue)

```
                         10        20        30        40        50        60
                 =========+=========+=========+=========+=========+=========+
Ec-06_004540     ----------MMDEDPLAAILGE-EEVHESEFLPGKNKGPKLNGGIDPEILAPDLLAACK
MSTRG.17397.1    WKSKKRQVLCLMDEDPLAAILGEKEELDDNNSRARKKNGSNGEDGTDPDILAPDLLAACK
                            ############            #########################


                         70        80        90       100       110       120
                 =========+=========+=========+=========+=========+=========+
Ec-06_004540     ENNDARAQDFLADGVPPGYSDPESGWSCLHWSVYNGNPMLTRALVRAGATSGYRRASNVL
MSTRG.17397.1    ENNVARAHDLLDDGAPPAFSDPTSGWSCLHWSVYNGNPVLTRALVRAGAATRYKRASTTL
                 ############################################################


                        130       140       150       160       170       180
                 =========+=========+=========+=========+=========+=========+
Ec-06_004540     TQGRKAHKQVNARRGPSSSLPISDTPLHWSAYRGDLGITWILLKEGYSPNDMDNMGNTAT
MSTRG.17397.1    MQGQKASKGNKARRAPKSSLPTTDTPLHWSAYRGDLGITWILLREGYSPNDTDNMGNTAA
                 ############################################################


                        190       200       210       220       230       240
                 =========+=========+=========+=========+=========+=========+
Ec-06_004540     HLAAANGHERVLDTLIRDGADVHRQNKFKNTPHDVANSAECRGILRRAEALPAPSVEEAE
MSTRG.17397.1    HLAAANGHERVLDTLIRDGADVHQQNKFKNTPYDVANSTECRNILKRAEALPPPSVEDAE
                 ############################################################


                        250       260       270       280       290       300
                 =========+=========+=========+=========+=========+=========+
Ec-06_004540     KMHTENLQRILEVERVLAEVVGPLEPTDDEDRRQKRKLQQLQGIKQAKDVDNRDDEELGE
MSTRG.17397.1    KMHHENLQRILEVERALIEAVGSLEPINDENRRRKRQMDEGGYARPGDDETAMDDDEEDN
                 ####################################                        


                        310       320       330       340       350       360
                 =========+=========+=========+=========+=========+=========+
Ec-06_004540     ARVGDEVTGGTVGAIATGGQAAGEGEGGGSDGGKGEDDPSNGGGENCQDSHGRHSAAEEK
MSTRG.17397.1    GGFQHKTS------------AEEGGEMT--------------------------------
                                                                             


                        370       380       390       400       410       420
                 =========+=========+=========+=========+=========+=========+
Ec-06_004540     TGEEGRMDAKET--DGDGGDDYKAGRTNNGDGGERRQGDVGIETHGTEGKEPDRGSRGSV
MSTRG.17397.1    ----GSADGKGVVVPGD-------------------------------GESP--------
                                                                             


                        430       440       450       460       470       480
                 =========+=========+=========+=========+=========+=========+
Ec-06_004540     GELSEDLLGQVELIREAVAGAEALCICEDLVAAARLRIDGLLLQHSIEIQATKVAAEGPI
MSTRG.17397.1    GE-----------------------------------------------KVARVGGDDS-
                                                                             


                        490       500       510       520       530       540
                 =========+=========+=========+=========+=========+=========+
Ec-06_004540     LTQQAYTTLVNRLMLLLRRAEGNSRISSKLKKRAKALVDKSHGEYWLQVSAQRLLKVECA
MSTRG.17397.1    ------------------RSKGDGDVSNQQNN----------------------------
                                                                             


                        550       560       570       580       590       600
                 =========+=========+=========+=========+=========+=========+
Ec-06_004540     EARHMADMAKLEARIEKAAKVEAHPQLVQDCRSLLKRLTTEVEISKAILGFPTVRLPIDP
MSTRG.17397.1    ------------------------------------------------------------
                                                                             


                        610       620       630       640       650       660
                 =========+=========+=========+=========+=========+=========+
Ec-06_004540     MPKDYWQPEDIGHIEENEGFPLPPVDADGKNMDYVWVPSKSLRALSAACKRMSEALVGGE
MSTRG.17397.1    ------------------------------------------------------HL----
                                                                             


                        670       680       690       700       710
                 =========+=========+=========+=========+=========+========
Ec-06_004540     GLNPALVERGSKKLQEFQKELRVLTEKDTADKDKAISVAAKAAKKKKKKGGGGKKKKK
MSTRG.17397.1    -------------------------------------------------------IGE
```

```
Parameters used
Minimum Number Of Sequences For A Conserved Position: 2
Minimum Number Of Sequences For A Flanking Position: 2
Maximum Number Of Contiguous Nonconserved Positions: 8
Minimum Length Of A Block: 10
Allowed Gap Positions: None
Use Similarity Matrices: Yes
```

```
Flank positions of the 2 selected block(s)
Flanks: [12  23]  [36  276]  

New number of positions in Ec-06_004540.aa.fasta.trans.aa_ali.fasta-gb:  253  (35% of the original 718 positions)
```
